# Comparison of Sufentanil- and Fentanyl-based Intravenous Patient-controlled Analgesia on Postoperative Nausea and Vomiting after Laparoscopic Nephrectomy: A Prospective, Double-blind, Randomized-controlled Trial

**DOI:** 10.7150/ijms.39374

**Published:** 2020-01-14

**Authors:** Hye-Mi Lee, Hae Keum Kil, Bon Nyeo Koo, Min Sup Song, Jin Ha Park

**Affiliations:** Department of Anesthesiology and Pain Medicine, and Anesthesia and Pain Research Institute, Yonsei University College of Medicine, Seoul, Republic of Korea.

**Keywords:** fentanyl, intravenous patient-controlled analgesia, laparoscopic nephrectomy, postoperative nausea and vomiting, sufentanil

## Abstract

**Background**: The incidence of postoperative nausea and vomiting (PONV) remains high. The effects of sufentanil for PONV is not firmly confirmed. The aim of this study was to compare the effect of sufentanil- and fentanyl-based intravenous patient-controlled analgesia (IV-PCA) on the incidence of PONV after laparoscopic nephrectomy.

**Methods**: Eighty-six patients were randomly allocated to receive either the sufentanil (n =43) or fentanyl (n =43). IV-PCA was prepared using either sufentanil 3 µg/kg or fentanyl 20 µg/kg, ramosetron 0.3 mg, and ketorolac 120 mg. The primary outcome of was the incidence of PONV during 24 h after post anesthesia care unit (PACU) discharge. The secondary outcomes were the modified Rhodes index and patient satisfaction scores at 24 h after PACU discharge, need for rescue antiemetics, pain score, need for additional analgesics, and cumulative consumption of IV-PCA

**Results**: The incidence of PONV was comparable between the sufentanil and fentanyl groups (64.3% vs. 65%, p = 0.946; respectively). The number of patients who required antiemetics (p = 0.946) and the modified Rhodes index at 24 h after post-anesthesia care unit discharge (p = 0.668) were also comparable in both groups. No significant differences were found in the secondary outcomes, including the analgesic profiles and adverse events between the groups.

**Conclusions**: In conclusion, sufentanil- and fentanyl-based IV-PCA showed similar incidence of PONV with comparable analgesic effects after laparoscopic nephrectomy. Based on these results, we suggest that sufentanil and fentanyl may provide comparable effects for IV-PCA after laparoscopic nephrectomy.

## Introduction

Intravenous patient-controlled analgesia (IV-PCA), which is based on strong opioids, is widely used for postoperative pain relief. Among these opioids, the three synthetic ones, alfentanil, fentanyl and sufentanil are the most common in anesthetic practice. Opioids act mainly on mu-receptors and exhibit powerful analgesic effects; however, inevitable side effects such as postoperative nausea and vomiting (PONV) are frequently encountered. Opioids are indeed a double-edged sword for PONV, because insufficient pain control after surgery itself is a risk factor for PONV while the incidence of PONV is dose-dependent with opioids.^1^,^2^

PONV decreases patient satisfaction, and may adversely affect prognosis by increasing wound dehiscence and dehydration, which may result in aspiration pneumonia and delayed recovery.^3^,^4^ The pathogenesis of PONV involves multiple pathways, including pain, anxiety, surgery-related factors and stimulation of the chemoreceptor trigger zone (CTZ) by anesthetics or opioids via neurotransmitters (e.g., dopamine, acetylcholine, histamine and serotonin). These factors often influence and/or overlap with each other, leading to PONV.^1^,^5^ To date, no single strategy has conclusively demonstrated an ability to prevent PONV.

Although laparoscopic surgery is considered advantageous due to the lower risk of postoperative pain and bleeding than open surgery, but it is classified as high-risk surgery for PONV due to the use of carbon dioxide (CO_2_) for pneumoperitoneum.^1^,^6^ In this subset of patients, the incidence of PONV has been reported to be as high as 50-75%.^1^,^6^ Thus, these high-risk patients might be targeted for PONV prevention by eliminating correctable risk factors, such as opioid dose reduction or proper pain management.^1^,^4^

Fentanyl is often used to control postoperative pain because of its relatively fast effect and short duration, whereas its incidence of PONV is relatively high.^7^,^8^ Sufentanil has a faster onset of action due to its higher affinity for mu-opioid receptors, lipophilic properties, and easier passage through the blood-brain barrier; therefore, it exhibits the most potent analgesic effect among clinically applied opioids.^9^-^12^ Additionally, although previous studies have yielded conflicting results, it is known that sufentanil is associated with a lower incidence of PONV than fentanyl due to its structural properties.^13^,^14^ However, limited data are available comparing these two drugs on the incidence and severity of PONV in patients undergoing laparoscopic nephrectomy who are at high risk for PONV. Therefore, we designed this double-blind randomized controlled study to compare the fentanyl and sufentanil-based IV-PCA on the incidence and severity of PONV in patients undergoing laparoscopic nephrectomy.

## Methods

This study was conducted at Severance Hospital, Yonsei University Health System, Seoul, Korea, approved by our institutional review board (IRB number, 4-2017-0201), and registered at http://www.ClinicalTrials.gov (NCT03171610). Written informed consent was obtained from each patient before study enrollment.

### Patients

Eighty-six patients, aged 20 to 70 years, with an American Society of Anesthesiologists (ASA) physical status I-III scheduled for elective laparoscopic or robot-assisted laparoscopic nephrectomy, and who required IV-PCA for postoperative pain control were enrolled. Patients were excluded if they met at least one of the following criteria: (1) long-term use of opioids, pain killers or tranquilizers; (2) a history of diabetic neuropathy; (3) a prolonged prothrombin time or an activated partial thromboplastin time; (4) cognitive function impairment; (5) obesity (body mass index ≥ 30 kg/m^2^); (6) conversion from a laparoscopic to open surgery; or (7) reoperation within 24 h.

Patients were randomly assigned to either sufentanil (n = 43) or fentanyl group (n = 43). Randomization was performed by computer-generated randomized numbers. For each patient, group assignments were kept in a sequentially numbered sealed, opaque envelope. An investigator who was not involved with outcome measurement and patient care opened the envelope on the day of surgery and prepared the IV-PCA according to the group assignment.

### Anesthetic management and Intervention

Premedication was not administered. After applying standard monitoring devices, general anesthesia was induced with propofol 2 mg/kg, remifentanil 1µg/kg, and rocuronium 0.8 mg/kg. Anesthesia was maintained with sevoflurane or desflurane, with 50 % oxygen in air, and remifentanil 0.1 to 0.2 µg/kg/min. The depth of anesthesia was controlled using a bispectral index (BIS) between 45 and 50. Propacetamol 15 mg/kg in total of 100 ml of 0.9 % normal saline was administered 20 min before the end of surgery, and fentanyl 1 µg/kg was injected 10 min before the end of surgery for postoperative pain prophylaxis. Ramosetron 0.3 mg was administered at the end of surgery for nausea prophylaxis, followed by continuous infusion of IV-PCA.

IV-PCA was prepared using either sufentanil 3 µg/kg or fentanyl 20 µg/kg, ramosetron 0.3 mg, and ketorolac 120 mg in 0.9% normal saline with a total volume of 100 ml according to the group assignment. We administered fentanyl and sufentanil with a potency ratio of 1:7, based on results from a previous study.^14^ The PCA device (Ambix anapa, Ewha Fresenius Kabi Inc., Gyeonggi-do, Korea) was programmed to deliver 2 mL/h as the background infusion with a 0.5 ml bolus at a 15-min lockout period.

### Assessments

After surgery, patients were asked to report their nausea and vomiting upon arrival at the post-anesthesia care unit (PACU); 30 min after arrival at PACU; 1, 6, and 24 h after discharge from the PACU on a binary scale (yes or no). The severity of pain at rest and coughing was also assessed at the same interval using a verbal numerical rating scale (VNRS), where 0 means no pain and 10 is the worst imaginable. Intravenous metoclopramide 10 mg was given as a rescue antiemetic at the patients' request. If severe nausea persisted after two consecutive rescue antiemetics, PCA was stopped for 2 h and restarted later when the symptoms subsided. Patients were allowed to receive intravenous pethidine 25 mg when they complained of pain >4 on VNRS. The Ramsay sedation score and cumulative consumption of IV-PCA were recorded at the same interval to evaluate postoperative sedation due to opioid use. The modified Rhodes index score 15 was assessed to evaluate the severity of PONV at 24 h after PACU discharge. Patients were asked about their satisfaction with IV-PCA for the previous 24 hours, using a scale with 0 meaning not satisfied to 10 meaning to very satisfied. Any adverse events, such as pruritis and dizziness, were evaluated. Patients were assessed by a trained nurse who was blinded to the group assignment.

The primary outcome of this study was the incidence of PONV 24 h after PACU discharge. The secondary outcomes were the modified Rhodes index and patient satisfaction scores at 24 h after PACU discharge, pain score, the need for rescue antiemetics or additional analgesics, and cumulative consumption of IV-PCA. The time to first flatus was also assessed.

### Statistical analysis

Sample size was calculated based on the previous studies that addressed the incidence of PONV in patients using fentanyl- or sufentanil-based IV-PCA for postoperative pain relief after various laparoscopic surgeries.^16^-^23^ The average incidence of PONV during 24 h were 39% and 17% during in the fentanyl and sufentanil groups, respectively. Based on these findings, we assumed a 20% difference in the incidence of PONV between the two groups. Therefore, 39 patients were required in each group to obtain a power of 80% with an alpha of 0.05. Considering a 10 % dropout rate, we decided to enroll 43 patients in each group.

Statistical analysis was performed using SPSS 25 (SPSSFW, SPSS, IBM, Armonk, NY, USA). Continuous variables were expressed as mean ± standard deviation (SD) or median [interquartile range] and were compared using Student's *t* test or the Mann-Whitney *U* test, as appropriate. Dichotomous variables were expressed as the number of patients (percentage) and were compared using Chi-square and or Fisher's exact test, as appropriate. For secondary analysis, Bonferroni correction was performed to compare inter-group differences of five time-points. P values of <0.05 were considered statistically significant.

## Results

**Figure 1:**
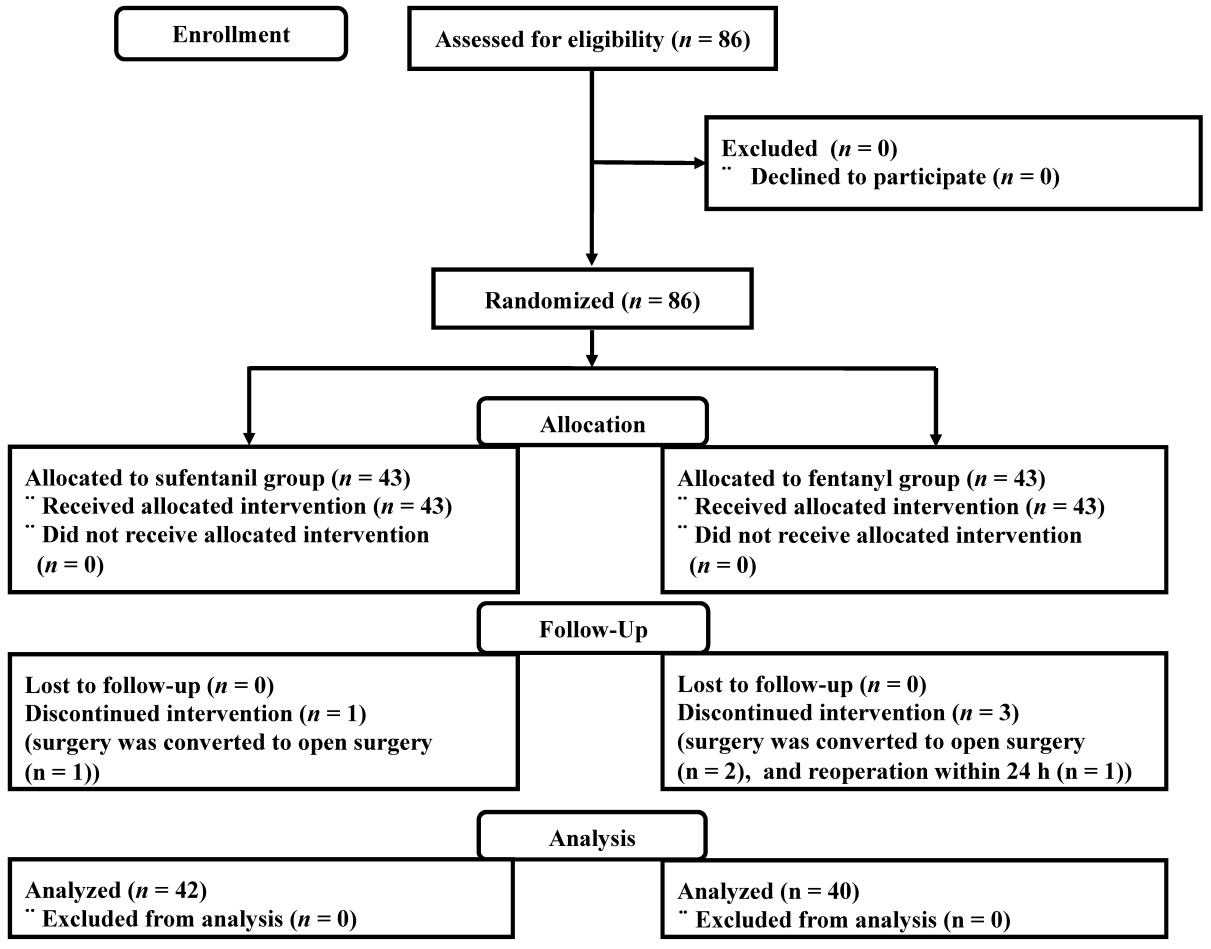
Consort flow diagram.

Between June 2017 and January 2018, a total of 86 patients were enrolled. One patient in the sufentanil group and two patients in the fentanyl group were excluded because surgery was converted to open surgery. One patient in the fentanyl group was excluded after the reoperation within 24 h due to postoperative bleeding (Figure 1).

Patients' demographic data were similar between the groups (Table 1). PONV occurred in 27 (64.3%) and 26 (65%) patients in the sufentanil and the fentanyl groups, respectively (p = 0.946) (Table 2). The number of patients who required antiemetics and the modified Rhodes index at 24 h after PACU discharge were comparable between the groups (Table 2).

No differences were observed in the postoperative analgesic profiles, including pain scores at rest and during coughing, the number of patients who required analgesics, cumulative consumed volume of IV-PCA, the number of patients who clamped the IV-PCA, and the patient satisfaction score between the groups (Table 3). There were no significant differences in postoperative adverse events and Ramsay sedation score (Table 4).

The number of patients who required antiemetics (26 vs. 3, p=0.001), stopped IV-PCA (36 vs. 7, p <0.001) and reported dizziness (37 vs. 10, p=0.003) were higher in patients with PONV than in those without PONV, respectively. Postoperative analgesic profiles, time to flatus, and patient satisfaction score were not significantly different between patients with PONV and without PONV (data not shown).

## Discussion

This prospective, double-blind, randomized controlled trial in patients undergoing laparoscopic nephrectomy showed no difference in the incidence of PONV between sufentanil-based and fentanyl-based IV-PCA. Additionally, the intensity of PONV and the postoperative pain profiles were comparable between the two treatment groups.

Despite many dedicated efforts to reduce the incidence of PONV, it still remains “*the big little problem”*, as Kapur described. 24 The mechanisms of PONV are multifactorial, and Apfel's risk factors for PONV include female gender, non-smoker, postoperative opioid use, and a history of PONV or motion sickness.^4^ Among these, postoperative opioid use is the only correctable factor; although, the use of opioids is inevitable after major surgery. Therefore, current strategies to manage and prevent PONV focus on the avoidance of higher doses of opioids and choosing an appropriate type of opioid as well as prophylactic administration of antiemetic drugs.^8^,^25^,^26^

Although there is still debate regarding whether the type of surgery contributes to the development of PONV, laparoscopic surgery has a potential to increase the incidence of PONV compared with open surgery due to the use of CO_2_ pneumoperitoneum, which results in peritoneal distension and visceral irritation.^27^ In a previous study, Yamanaga et al. reported an incidence of PONV higher than 60% in patients undergoing laparoscopic donor nephrectomy.^28^ Therefore, an opioid-based PCA regimen with a lower risk of PONV is particularly important among high-risk laparoscopic surgery patients.

In this present study of patients undergoing laparoscopic nephrectomy, the incidence of PONV at 24 h after PACU discharge was 64.3% in the sufentanil group and 65% in the fentanyl group without significant difference. A possible explanation for this high incidence of PONV is that more than 50% of patients had Apfel scores of 3 or 4 in both groups. In a previous study comparing sufentanil- and fentanyl-based IV-PCA after lumbar fusion, the incidences of PONV was 4.76% and 28.5% in the sufentanil and fentanyl groups, respectively, even though they used higher doses of sufentanil (4 µg/kg) and fentanyl (24 µg/kg) than those in this study.^14^ Considering that Kim et al and Choi et al reported approximately 70% incidence of PONV in highly susceptible patients undergoing lumbar surgery,^29^,^30^ while Park et al reported approximately 30% incidence with a similar dose of fentanyl-based IV-PCA in patients at moderate risk undergoing lumbar surgery,^31^ the PONV risk itself, in other words, individual patients' susceptibility may be important in the development of PONV. 32 Furthermore, a lower incidence of PONV in the sufentanil group than that in the fentanyl group in the Kim et al's study 14 was possibly due to the lower proportion of females, consequently leading to a lower risk of PONV.

Numerous previous studies comparing sufentanil and fentanyl have focused on intrathecal injection, epidural PCA, and intraoperative infusion.^8^,^33^,^34^ Few studies have compared the effect of sufentanil- and fentanyl-based IV-PCA on the relative emetic potencies. Opioid-induced nausea and vomiting occur via stimulation of the CTZ or the vestibular apparatus, mediated by activation of the opioid mu receptor.^35^,^36^ Notably, the CTZ is located outside the blood-brain barrier and contains a high concentration of opioid receptors. Therefore, the CTZ is susceptible to opioid stimulation.^36^ Sufentanil has an 8-10 times higher lipid solubility than fentanyl, with a higher affinity for the mu-opioid receptor, and 5-10 times more potency than fentanyl; thus, smaller doses are required to achieve an analgesic effect. 11 Because the opioid-induced PONV risk increases in a dose-dependent manner,^2^ it has been suggested that sufentanil-based IV-PCA provide a powerful analgesic effect with a lower incidence of PONV than fentanyl-based IV-PCA.

Contrary to our expectation, the incidence of PONV was similar between the groups. The intensity of PONV reported by the modified Rhodes index at 24 h after surgery, the number of patients who required rescue antiemetics was also comparable throughout the study period. Because pain increases the incidence of PONV, we compared pain profiles, and there were no differences in pain profiles between the groups. The incidences of other opioid-related side effects, including dizziness and pruritis, were similar, and no patients reported excessive sedation in each group. Based on the results that there were no differences in all comparative values between the two groups, we suggest that the sufentanil and fentanyl dose ratio used in this study (1: 7) seems to be clinically equivalent.

It is noteworthy that the PCA satisfaction score and pain profiles were similar between patients with and without PONV; although, more patients with PONV stopped IV-PCA and required antiemetics. Therefore, this regimen of IV-PCA seems to be adequate for pain control in laparoscopic nephrectomy. Because ketorolac is also added to lower the opioid dose, other methods, such as adding two or more antiemetics with different mechanisms of action, should be considered to lower the incidence of PONV in moderate- to high-risk patients.^37^ Alternatively, considerably lower opioid doses may be needed for pain relief based on the fact that mutant variant of mu-opioid receptor genes is more sensitive and prevalent in Asian populations.^38^ However, this is beyond the scope of this study and will not discussed further here.

This study has limitations. The severity of PONV, which was evaluated by the modified Rhodes index, was assessed only at 24 h after PACU discharge. Although the incidence of PONV did not differ at each time point, there is a possibility that the severity of PONV might be different between the groups at different time point. However, we estimated the severity of PONV through the incidence of PONV and the number of patients who required antiemetics at each time point.

In conclusion, sufentanil-based and fentanyl-based IV-PCA showed a similar incidence of PONV with comparable analgesic effects after laparoscopic nephrectomy. Based on these results, we suggest that sufentanil and fentanyl may provide comparable effects for PCA after laparoscopic nephrectomy.

## Figures and Tables

**Table 1:** Demographic data

	sufentanil group (n = 42)	fentanyl group (n = 40)	P value
Age (yr)	52 ± 9	49 ± 12	0.291
Height (cm)	168 ± 8	165 ± 10	0.412
Weight (kg)	67 ± 11	68 ± 11	0.812
Female	15 (35.7%)	17 (42.5%)	0.651
Apfel score	2.5 [2-3]	2.5 [2-3]	0.959
Laparoscopic/Robotic surgery	12/30	12/28	>0.999
ASA I/II/III	21/19/2	23/15/2	0.809
Hypertension	12 (28.6%)	11 (27.5%)	>0.999
Diabetes	3 (7.1%)	4 (10%)	0.709
Anesthesia time (min)	213 [189 - 241]	195 [164 - 224]	0.168

Data are presented as mean ± standard deviation or median (interquartile range) and numbers (%). ASA, American Society of Anesthesiologists.

**Table 2:** The incidence of postoperative nausea and vomiting, modified Rhodes index, and use of rescue antiemetics.

	sufentanil group (n = 42)	fentanyl group (n = 40)	P value
Incidence of nausea/vomiting			
Overall	27 (64.3%)/ 4(9.5%)	26 (65%) / 4(10%)	0.946/ >0.999
PACU arrival	5 (11.9%)/0 (0%)	4 (10%)/0 (0%)	>0.999/-
30 min at PACU	2 (4.8%)/0 (0%)	4 (7.5%)/0 (0%)	0.672/-
1 h after PACU discharge	9 (21.4%)/0 (0%)	11 (27.5%)/0 (0%)	0.522/-
6 h after PACU discharge	11 (26.2%)/3 (7.1%)	12 (30.0%)/ 2 (5%)	0.701/ >0.999
24 h after PACU discharge	23 (54.8%)/ 4 (9.5%)	17 (42.5%)/ 4 (10%)	0.267/ >0.999
Number of patients who required antiemetics			
Overall	15 (35.7%)	14 (35%)	0.946
PACU arrival	4 (9.5%)	3 (7.5%)	>0.999
30 min at PACU	2 (4.8%)	0 (0%)	0.494
1 h after PACU discharge	5 (11.9%)	4 (10%)	>0.999
6 h after PACU discharge	6 (14.3%)	7 (17.5%)	0.690
24 h after PACU discharge	7 (16.7%)	6 (15%)	0.836
Modified Rhodes index	4 [0-9]	3 [0-8]	0.668

Data are presented as numbers (%). PACU, postoperative anesthesia care unit.

**Table 3:** Postoperative analgesic profiles

	sufentanil group (n = 42)	fentanyl group (n = 40)	P value
VNRS of pain (at rest/ during coughing)			
PACU arrival	6 [3-7]/ 7 [5-8]	5 [4-7]/ 6 [5-8]	0.428/0.594
30 min at PACU	5 [3-5]/ 6 [4-7]	4 [4-5]/ 5 [4-6]	0.569/0.507
1 h after PACU discharge	5 [4-7]/ 7 [5-8]	5 [2-6]/6 [5-8]	0.428/0.403
6 h after PACU discharge	2 [0-4]/ 4 [2-6]	3 [1-4]/ 4 [3-8]	0.475/0.858
24 h after PACU discharge	1 [0-2]/ 4 [2-4]	1 [0-3]/ 4 [2-5]	0.614/0.839
Number of patients who required analgesics			
Overall	30 (71.4%)	28 (70%)	>0.999
PACU arrival	24 (57.1%)	17 (42.5%)	0.269
30 min at PACU	6 (14.3%)	5 (12.5%)	>0.999
1 h after PACU discharge	13 (31%)	10 (25%)	0.627
6 h after PACU discharge	5 (11.9%)	7 (17.5%)	0.543
24 h after PACU discharge	5 (11.9%)	7 (17.5%)	0.543
Cumulative volume of IV-PCA consumed (mL)			
PACU arrival	4 [2-5]	5 [3-6]	0.891
30 min at PACU	5 [4-8]	6 [4-8]	0.799
1 h after PACU discharge	10 [6-15]	9 [7-12]	0.962
6 h after PACU discharge	32 [21-42]	35 [20-40]	0.919
24 h after PACU discharge	56 [38-65]	50 [40-60]	0.688
Number of patients who clamped the IV-PCA	22 (52.4%)	21 (52.5%)	>0.999
Patient satisfaction score	4 [3-4]	4 [3-5]	0.394

Data are presented as median (interquartile range) and numbers (%). IV-PCA, intravenous patient-controlled analgesia; PACU, post-anesthesia care unit; VNRS, verbal numerical rating scale.

**Table 4:** Postoperative adverse events and Ramsay sedation score

	sufentanil group (n = 42)	fentanyl group (n = 40)	P value
Time to flatus (h)	18 [12-27]	19 [14 - 23]	0.950
Dizziness	25 (59.5%)	22 (55%)	0.824
Pruritis	0 (0%)	1 (2.5%)	0.488
Ramsay sedation score			
PACU arrival	2 [1-2]	2 [2-2]	0.058
30 min at PACU	2 [1-2]	2 [2-2]	>0.999
1 h after PACU discharge	2 [1-2]	2 [2-2]	0.503
6 h after PACU discharge	2 [1-2]	2 [2-2]	>0.999
24 h after PACU discharge	2 [1-2]	2 [2-2]	>0.999

Data are presented as median (interquartile range) and numbers (%). PACU, post-anesthesia care unit

## Abbreviations

ASA: american society of anesthesiologists
BIS: bispectral index
CO_2_: carbon dioxide
CTZ: chemoreceptor trigger zone
IV-PCA: intravenous patient-controlled analgesia
PACU: post-anesthesia care unit
PONV: postoperative nausea and vomiting
VNRS: verbal numerical rating scale
